# Molecular Pathology of Lewy Body Diseases

**DOI:** 10.3390/ijms10030724

**Published:** 2009-02-26

**Authors:** Katrin Beyer, Montserrat Domingo-Sàbat, Aurelio Ariza

**Affiliations:** Department of Pathology, Hospital Universitari Germans Trias i Pujol, Autonomous University of Barcelona, Badalona 08916, Barcelona, Spain; E-Mails: montse.domingo.sabat@gmail.com (M.S.); aurelio.ariza@uab.cat (A.A.)

**Keywords:** Lewy body diseases, Parkinson disease, dementia with Lewy bodies, alpha-synuclein, molecular chaperones, mitochondrial dysfunction, proteosomal dysfunction, alternative splicing, differential isoform expression

## Abstract

Lewy body diseases are characterized by the presence of Lewy bodies, alpha-synuclein(AS)-positive inclusions in the brain. Since their main component is conformationally modified AS, aggregation of the latter is thought to be a key pathogenic event in these diseases. The analysis of inclusion body constituents gives additional information about pathways also involved in the pathology of synucleinopathies. Widespread mitochondrial dysfunction is very closely related to disease development. The impairment of protein degradation pathways, including both the ubiquitin-proteasome system and the autophagy-lysosome pathway also play an important role during the development of Lewy body diseases. Finally, differential expression changes of isoforms corresponding to genes primarily involved in Lewy body formation point to alternative splicing as another important mechanism in the development of Parkinson’s disease, as well as dementia with Lewy bodies. The present paper attempts to give an overview of recent molecular findings related to the pathogenesis of Lewy body diseases.

## Prologue

Lewy body diseases (LBD) share alpha-synuclein (AS) aggregation and Lewy body (LB) formation as their key pathogenic events. The determination of the molecular pathways that lead to AS oligomerization and further aggregation is the basis for the successful design and development of treatments for important neurodegenerative diseases such as Parkinson’s disease (PD) and dementia with Lewy bodies (DLB). After an introduction of the various LBD, this review summarizes molecular findings over the last two years that provide new insight into the pathogenesis of LBD.

## 1. Lewy body diseases

LBD are characterized by the presence on intraneuronal proteinaceous inclusions called Lewy bodies (LBs) with AS as their main component. Whereas these are found mainly within the brainstem in the case of PD, their widespread distribution through almost all brain areas is a characteristic feature in DLB. Neuropathologically, the presence of LB is accompanied by neurodegeneration in the affected areas, therefore the brainstem affectation in PD causes parkinsonian symptoms and the additional cortical affectation in DLB, dementia.

### 1.1. Parkinson’s Disease

First described in 1817, PD is the most common progressive movement disorder in the elderly and is characterized by tremor, rigidity, and bradykinesia. There is increasing evidence that PD is a multi-systemic disorder showing both progressive degeneration of the dopaminergic nigrostriatal system and widespread extranigral pathology [1–3]. In PD, LB pathology first appears in lower brainstem nuclei such as the dorsal motor nucleus of the vagus and the olfactory system (Stages 1–2). Afterwards, ascending progression leads to changes in the coeruleus complex, substantia nigra pars compacta, basal forebrain magnocellular nucleus, subthalamic nucleus, and amygdala (Stages 3–4). Finally, involvement of the neocortex may supervene (Stages 5–6) [1,2].

Over the last few years advances in PD genetics have revealed that mutations are responsible for only a small proportion of cases, the majority being of sporadic origin. Of the six genes responsible for Mendelian forms of PD, the first identified was the AS (SNCA) gene, in which three pathogenic point mutations (A30P, E476K and A53T) as well as duplications and triplications have been detected [3–7]. Genes involved in PD genetics by mutations in autosomic dominant familial cases the ubiquitin C-terminal hydrolase L1 (UCH-L1) gene [8], the dardarin gene, leucine-rich kinase 2 (LRRK 2) [9] and the HtrA2/Omi gene [10]. DJ-1 gene [11], PTEN-induced putative kinase 1 (PINK1) gene [12] and parkin (PRKN) gene mutations are responsible for autosomal recessive Parkinson cases [13]. Interestingly, the vast majority of PD instances associated with PRKN mutations lack LBs [14]. Sporadic PD has been associated to mutations in the synphilin [8], LRRK 2 [9] and HtrA2/Omi [10] genes and to the S18Y polymorphism of the UCH-L1 gene that lowers the risk to suffer PD [15].

### 1.2. Dementia with Lewy Bodies

DLB is the second most frequent cause of dementia in the elderly after Alzheimer disease (AD) [16] and is clinically characterized by progressive dementia, often accompanied by parkinsonism and psychiatric symptoms [17]. Widespread distribution of LBs in virtually every brain area is a typical feature of DLB, although the frontal cortex, pigmented midbrain and brainstem nuclei, dorsal efferent nucleus of the vagus, basal forebrain nuclei, and limbic cortical regions are particularly involved [18].

A high percentage of DLB cases show, in addition to LB related pathology, AD characteristic changes [19–21], where higher Braak stages of AD-type pathology result in a clinical diagnosis of AD rather than DLB [22]. Accordingly, the misdiagnosis of DLB increases with increasing Braak stages of AD associated pathology [23].

Four mutations, the E46K mutation on SNCA [5], the UCH-L1 gene I93M mutation [8] and two beta-synuclein mutations (V70M and P123H) [24] have been described in four DLB pedigrees. In contrast, up to the present, no genetic marker has been found to be associated with sporadic DLB.

## 2. Lewy Bodies

Similar to other neurodegenerative disorders, increasing evidence suggests that the presence of compact inclusions may represent a protective mechanism on the part of surviving cells [25,26]. Whereas LBD are characterized by the general neuronal loss of the dopaminergic system, a high percentage of surviving neurons contain intracellular inclusions in the form of LB. These seem to protect the cell by the up-take of misfolded and un-functional proteins [27,28].

### 2.1. Structure

Morphologically, LBs may be divided into brainstem and cortical types. Whereas brainstem LBs are found in the brainstem nuclei and diencephalon, cortical LBs are preferentially seen in the cerebral limbic cortex and amygdala. Classic brainstem LBs are spherical intraneuronal cytoplasmic inclusions, characterized by hyaline eosinophilic cores, concentric lamellar bands, narrow pale halos, and immunoreactivity for AS and ubiquitin [29]. In contrast, cortical LBs typically lack a halo [30]. AS immunolabeling of PD brains have revealed that AS immunostaining can be detected in approximately 64% of nigral LBs and 31% of cortical LBs [31].

### 2.2. Components

Up to the present, a large number of proteins has been identified in LBs, but their precise biochemical composition has not yet been elucidated [32].

The main component of inclusion bodies in synucleinopathies is AS and after its initial aggregation, additional proteins are captured and accumulated to the LB. More than 76 LB components have been described so far [33]. They belong to ten different protein classes, including structural elements, AS-binding proteins, synphilin-1-binding proteins, components of the ubiquitin-proteasome system, proteins implicated in cellular responses, proteins associated with phosphorylation and signal transduction, cytoskelatal proteins, cell cycle proteins, cytosolic proteins and others [33].

### 2.3. Formation

AS aggregation is now accepted as the key step preceding LB formation. Although the precise sequence of events responsible for AS fibrillation remains unknown, advances in elucidating the course of AS aggregation have been attained. First, aggregated AS species with altered solubility as well as substantial accumulation of detergent-soluble and detergent-insoluble AS species of various molecular weights are found particularly in the grey matter [29]. As revealed by ultrastructural studies of AS filaments in LBs, pale bodies, and perikaryal threads, AS perikaryal threads are an early stage of AS filament assembly [34]. Pale bodies occur after incorporation of p62 and assimilation of less aggregated forms of AS [30]. Afterwards, LB appearance would be coincidental with increasing ubiquitin immunoreactivity, due to further incorporation of mono- and di-ubiquitylated AS [35] and capture and [36] accumulation of additional proteins [36,37]. Final stages of LB formation are characterized by involvement of dendrites, LB distortion and finally by LB degradation showing loose filamentous components, decreased AS immunoreactivity, and astroglial process involvement [38].

## 3. Alpha-synuclein oligomerisation and aggregation – neurotoxicity vs. protection

AS aggregation represents a key event in the pathogenesis of LBD. Therefore, AS structure, conformational behaviour and aggregatory properties have been extensively studied over the last years. Accumulated knowledge can be consulted in many review articles concerning this topic [39,40–45].

AS aggregation is preceeded by its oligomerization. But in addition to oligomers that undergo fast transition to a fibrillary state, stable prefibrillar AS oligomers are also detected. These AS protofibrils constitute the deleterious species of the protein [46,47] and are toxic, causing cell death. Specifically, AS 67–82, 72–82 and 72–80 peptides, located within the central or NAC region, display in addition to their toxicity, beta-sheet secondary structure and aggregate forming fibrils [48–50]. Three possible mechanisms for AS neurotoxicity have been proposed. First, direct AS interaction with histones inhibiting their acetylation could cause nuclear AS neurotoxicity [51,52], second, AS protofibrils with pore-like activity or with pore-like structure, are able to permeabilize membranes, so disrupting ionic and metabolic homeostasis [45,53] and third, AS was shown to inhibit the function of the neuronal survival factor MEF2D causing about 40% loss of neuronal viability [54].

In contrast, inclusion body formation does not necessarily lead to cell death, but seems to represent a protective mechanism of the cell for survival [25,26]. Nevertheless, it has not been excluded that AS aggregates per se or some event associated with the AS aggregation process are neurotoxic [55]. On one hand, cell and animal models of different neurodegenerative diseases develop inclusion bodies that conversely correlate with cell death [56], and synphilin-1 and synphilin-1A inclusion bodies can be cytoprotective in PD [8,25,57,58]. However, on the other hand, inclusions formed by monoubiquitylated AS increse cell death, and AS inclusions in Drosophila are neurotoxic [59]. Moreover, the fact that monoubiquitylated AS forms amorphous cytotoxic inclusions [35] and that LBs contain amourphous aggregated proteins in the core and fibrils in their periphery [60], imply that LB may be toxic to cells at their initial stages of formation [35]. The further co-aggregation of additional PD-related proteins may counteract this toxicity that is possibly present at initial stages. Nevertheless, to date it is still impossible to predict how LB formation affects dopaminergic neuron viability in PD patients because of their complex protein composition [61].

Ubiquitylation and phosphorylation of both AS and AS aggregation related proteins, are important mechanisms in the regulation of AS aggregation. Whereas about 10% of AS is monoubiquitilated in LBs [62,63], soluble AS is not [63]. Although AS monoubiquitylation rates are low within LBs, these could enhance AS aggregation and seed the formation of inclusion bodies [61].

Parkin, UCH-L1 and PINK1 are also proteins found in LBs [33]. When ubiquitylated, all of them are able to form intracellular inclusions [64,65], indicating that ubiquitylation of different proteins involved in PD may contribute to LB formation [66].

Phosphorylation modulates the ubiquitylation and aggregational properties of various proteins [67]. In addition to being monoubiquitylated, AS is also phosphorylated at serin 129 in LBs [63,68]. Moreover, protein kinases, such as GSK3ß and Cdk5 modulate the death of dopaminergic neurons in pharmakological PD models [69,70]. Of these, Cdk5 phosphorylates parkin at serine 131, decreasing its ability to ubiquitylate synphilin-1 [71]. Parkin moreover decreases synphilin-1 and AS toxicities and promotes inclusion body formation [72].

Synphilin-1, an AS interacting protein, interacts with and is phosphorylated by the protein kinases GSK3ß and casein kinase II (CKII) [73,74]. Whereas GSK3ß decreases synphilin-1 ubiquitylation and inclusion body formation [74], CKII increases inclusion body formation regardless of synphilin-1 ubiquitylation [73,74].

The exhaustive analysis of LB components in dependence on the disease stage may give information about the dysfunction of the various pathways involved in LB disease development.

It is worth mentioning that in addition to AS, all other proteins linked to PD or DLB by disease causing mutations, are also found in LBs. Of these, PINK1, parkin, DJ-1 and HtrA2/Omi are mitochondria related genes, UCH-L1 and also parkin proteasome related proteins, and synphilin-1 has been related to proteosomal dysfunction by its ablity to interact with various proteosomal proteins. Beta-synuclein is an exception, because although mutated in DLB it is not found in LBs, probabely because of its AS-antiaggregatory properties.

## 4. Mitochondrial dysfunction

Mitochondrial dysfunction, in part thought to be responsible for oxidative stress, is an important factor involved in the preferential cell loss seen for some groups of neurons in the brain [75]. Accordingly, some of the genes that cause recessive parkinsonism are associated, directly or indirectly, with mitochondrial function [76–78]. Although recessive parkinsonism differs phenotypically from LBD, in addition to the common feature of prominent nigral cell loss, an important role of mitochondrial dysfunction also in LBD has been suggested. Two of such PD and mitochondrial function related proteins are parkin and PTEN-induced kinase 1 (PINK1) [79,80].

### 4.1. Parkin

Parkin is an ubiquitin E3 ligase that contains a ubiquitin like domain, two ring finger domains and between the ringfinger domains, a conserved region [81]. As ubiquitin E3 ligase, parkin has been related to the proteasome, but it has not been excluded that this activity can be proteasome independent [80].

Parkin loss-of-function Drosophila models exhibit dramatic defects of mitochondrial morphology [82,83] and parkin knockout mice show signs of oxidative tissue damage in the brain [84]. Although initially several fly and mouse models have located parkin partially in the outer mitochondrial membrane, at steady state it is primarily cystosolic [84–86] and it participates in the regulation of mitochondrial function [84,85].

Together with PINK1 parkin constitutes the Pink1/parkin pathway that promotes mitochondrial fission [87,88]. PINK1 could act through a signal transduction cascade promoting parkin dependent ubiquitylation of particular cytoplasmic targets related to mitochondrial morphogenesis [87]. Moreover, parkin is strikingly and specifically recruited to dysfunctional mitochondria, where it regulates their selective removal by autophagy [86,89].

### 4.2. PINK-1

The PTEN-induced putative kinase, PINK1 localizes predominantly within mitochondria, specifically at the inner mitochondrial membrane [90,91].

PINK-1 loss-of-function Drosophila presents reduced ATP levels as well as mitochondrial DNA content [77,78,92] and mitochondria within dopaminergic neurons present an altered morphology [78]. Since parkin overexpression in flies suppresses all PINK1 mutant phenotypes, but PINK1 overexpression does not compensate for loss of parkin function [77,78,92], PINK1 and parkin seem to act in a linear pathway that affects mitochondrial function with parkin downstream to PINK1 [80]. In peripheral tissues from PD patients with PINK1 [93] and parkin [94] mutations, as well as with sporadic PD [95] have been detected pathological changes and defects in mitochondrial respiration.

Furthermore, PINK1 directly phosphorylates parkin [96] but also the mitochondrial serin protease Omi/HtrA2 [97]. Phosphorylation of parkin induces its translocation to mitochondria so that PINK1 mutations could cause PD by decreased PINK1 kinase activity that at the same time would lead to low parkin levels within mitochondria [96].

### 4.3. Omi/HtrA2

Omi/HtrA2 contributes to the progression of apoptosis [77,78,98,99]. It is released from the mitochondrial intermembrane space into the cytosol, where it induces apoptotic cell death in addition to the permeabilization of the mitochondrial membrane that at the same time leads to cytochrome c release [98–100].

Although Omi/HtrA2 is phosphorylated in a PINK1-dependent manner [97], the functional importance of this interaction remains to be elucidated. However, both PINK1 and Omi/HtrA2 interact and Omi/HtrA2 functions downstream of Pink1 [101]. Surprisingly, there is no evidence of interaction between Omi/HtrA2 and parkin, and both act as independent downstream effectors of PINK1. Although emerging evidence indicates that the PINK1/Parkin pathway is likely to affect mitochondrial dynamics, the function of the PINK1/Omi/HtrA2 pathway is currently unclear [101].

Similar to parkin and PINK1, both the Omi/HtrA2 S399 mutant and the Omi/HtrA2 A141S polymorphism, induce mitochondrial dysfunction associated with altered mitochondrial morphology [102].

## 5. Dysfunction of protein degradation pathways

The ubiquitin-proteasome system (UPS) and autophagy-lysosome pathway (ALP) are the two most important mechanisms that normally repair or remove abnormal proteins from the cell. Alterations in the function of these systems to degrade misfolded and aggregated proteins are being increasingly recognized to play a fundamental role in the pathogenesis of many neurodegenerative disorders.

If AS degradation depends on the UPS or ALP remains unknown, but both seem to be involved in this process [103]. The recent finding that the inhibition of autophagy leads to the accumulation of monoubiquitilated AS forms followed by its aggregation [61], indicates that autophagy could be the predominant pathway involved in AS clearance [35,61].

### 5.1. Proteasomal dysfunction

The first indication for UPS dysfunction in diseased neurons was the detection of ubiquitylated proteins in proteinaceous inclusions found in neurodegenerative diseases [104]. Now it is known that polyubiquitylation is the canonical signal for targeting proteins to the proteasome for degradation [67]. Monoubiquitilation, on the contrary, regulates different cellular functions such as protein sorting in the late endosomal pathway, histone function and DNA repair [105].

The involvement of the UPS dysfunction in PD was further underlined by the finding of decreased proteasome activity in sustancia nigra of PD brains [106]. Parkin, not only involved in mitochondrial function and dysfunction, is together with UCH-L1, a component of the UPS. Some familial PD forms are caused by mutations in their genes [107], and whereas parkin polyubiquitilates a rare glycosylated AS form [108], UCH-L1 polyubiquilates AS in vitro [109].

#### 5.1.1. UCH-L1

Linked to the UPS, the ubiquitin C-terminal hydrolase UCH-L1 is an abundant neuronal protein that moreover stabilizes free ubiquitin [110]. A missense mutation, I93M, has been detected in a German family with PD [107], and the S18Y polymorphism was found to be protective against PD in different populations [111,112]. Whereas the I93M mutant decreases UCH-L1 hydrolase activity by 50% [107], the protective S18Y variant results in a slight increase of hydrolase activity [113].

Moreover, it has been recently shown that the UCH-L1 S18Y variant acts as a potent antioxidant [110], protecting cells from induced oxidative damage in different cell models [110]. Its overexpression leads to protein aggregation, but not to apoptosis [110].

#### 5.1.2. SIAH-1

SIAH-1, along with parkin and UCH-L1, belongs to the UPS and is also an E3 ubiquitin ligase that has been detected in LB of PD patients [57]. SIAH-1 regulates the ubiquitilation and degradation of synaptophysin [114] and its interaction with synphilin-1 [57,58,71] is followed by synphilin-1 polyubiquitylation and proteosomal degradation [115].

Furthemore, SIAH-1 seems to play a critical role in the regulation of AS aggregation and toxicity [66]. In contrast to polyubiquitylation of synaptophysin and synphilin-1, SIAH-1 interacts with and mono- and di-ubiquitylates AS [55,61]. AS purified from LB is ubiquitylated at lysines 12, 21, and 23 [63].

#### 5.1.3. Synphilin-1

Synphilin-1 was originally identified as an AS interacting protein [116] and its co-expression with AS in cell cultures leads to the formation of LB-like inclusions [73,116,117].

Even if synphilin-1 is not located within the UPS, it is able to interact with various UPS related proteins. As a 919 amino acid protein, synphilin-1 contains different domains such as ankyrin-like repeats, a coiled-coil domain and a putative ATP,GTP-binding domain [116]. Moreover, synphilin-1 localizes in the presynapse where it binds to synaptic vesicles [117] and may affect dopamine release [115].

The first evidence that synphilin-1 interferes with the proteosomal function was that synphilin-1 overexpression decreases of the reporter protein (GFPu) degradation, a protein normally highly degraded by the proteasome [74]. In addition, synphilin-1 interacts with two proteasome subunits/regulators, S6 ATPase (tbp7) and NUB1 [66]. Its interaction with S6 decreases proteasome function on one hand and co-expression of synphilin-1 and the S6 subunit increases the formation of aggresome-like intracytoplasmic inclusions, on the other [118]. Both not only co-localize in these inclusions, but also in LBs in PD [118].

Finally, synphilin-1 interacts with three E3 ubiquitin ligases: parkin, SIAH, dorfin. Whereas both parkin and SIAH ubiquitylate synphilin-1, followed by inclusions body formation, non-ubiquitylated synphilin-1 is not able to form inclusions [57,74,117,119].

Because of its main characteristic to interact with multiple proteins and many of these are directly related to LB diseases, it has been proposed that synphilin-1 may assemble these proteins into a multi-protein complex [66]. Accordingly it is located within the core of brainstem LB, but is also detected in cortical LB [33].

### 5.2. Dysfunction of the autophagy-lysosomal pathway

ALP comprises three distinct pathways based on the ways substrates reach the lysosomal lumen: macroautophagy, or autophagy, microautophagy and chaperone-mediated autophagy (CMA) [120]. Of these, CMA selectively degrades cytosolic proteins [121]. In contrast to the UPS, autophagy seems to be the primary mechanism involved in the degradation of long-lived, stable proteins. Moreover, it is the only mechanism by which entire organelles such as mitochondria and large membrane proteins and protein complexes are recycled [120,122].

Evidence for the involvement of lysosomal dysfunction in LBD has been obtained during the last years. Deficiency of the lysosomal enzyme cathepsin D leads to the accumulation of endogenous AS in neurons without the overabundance of its mRNA. Conversely, cathepsin D overexpression reduces AS aggregation and shows a neuroprotective effect against AS overexpression-induced cell death in vitro [123]. Moreover, wild-type AS, but not mutant AS, is selectively translocated into lysosomes for degradation by the CMA pathway [120]. Mutant AS show a very increased affinity to lysosomal membrane receptors, a fact that could explain the failure of CMA to clear it [120].

Finally, very recently, lysosomal dysfunction has been directly related to AS overexpression and accumulation. A study on the regulation of the myocyte enhancer factor 2D (MEF2D), a neuronal survival factor, revealed that AS disrupts MEF2D CMA-mediated degradation [54]. MEF2D, normally located within the neuronal nucleus [121], translocates to the cytoplasm where it is degraded by the lysosomal system [54]. Overexpression of both wild type and mutated AS, inhibits the uptake of MEF2D by lysosomes and its degradation.

## 6. Alternative splicing

Alternative splicing is a versatile and widespread mechanism for the generation of multiple mRNAs from a single transcript. It leads to significant changes in protein secondary structure and these modifications may cause functional alterations by the specific deletion of functional protein domains. In the last few years an increasing number of genes involved in neurodegenerative disorders have been shown to express more than one splice variant differentially.

Examples include the presenilin (PS) genes (PS1 and PS2), both of which undergo alternative splicing. Of the PS1 isoforms, one lacks exon 9 and the other two variably incorporate four amino acids (VRSQ) on exon 4 [124]. The four amino acid VRSQ-lacking isoform binds to the RabGDP dissociation inhibitor (rabGDI), whereas the VRSQ-containing isoform does not, a finding that constitutes the first evidence of a specific function for the different isoforms [125]. On the other hand, one of PS2 transcript variants lacks exon 5, resulting in a short, truncated protein which is differentially overexpressed in AD [126].

Another alternatively spliced gene is tau, of which six isoforms resulting from complex splicing events are expressed in the adult brain [127–129]. The inclusion of exon 10, which codes for an additional microtubule-binding domain, causes frontotemporal dementia with parkinsonism [130]. Finally, it has been recently shown that differential expression of parkin and synphilin transcript variants is altered in LBDs [57,131].

### 6.1. AS alternative splicing

Thus, four AS isoforms that result from alternative splicing have been described [39,132]. In addition to AS 140, representing the whole and major transcript of the protein [132–134], three other isoforms also exist: AS 126, AS 112 and AS 98. Whereas inframe deletions of exons 3 or 5 give rise to AS 126 and AS 112, respectively [133,134], the deletion of both exons 3 and 5 is found in AS 98 [132]. Exon 3 encodes for residues 41–54 within the N-terminus and its deletion results in the interruption of the four amphipathic α-helices of the membrane-binding domain. In contrast, exon 5 encodes for residues 103–130 within the C-terminus, and its splice out shortens the unstructured AS part. Finally, AS 98 mainly preserves the central region of the protein intact. These structural changes resulting from alternative splicing of AS RNA strongly indicate functional changes of the shortened protein isoforms [132,135,136].

#### 6.1.1. AS 126

AS 126 could represent an AS isoform with diminished aggregation properties due to the interruption of the membrane binding domain thought to be necessary during the first steps of AS aggregation [137]. In this context, we have shown that a polymorphic polyT site located in intron 2, co-regulates AS 126 expression levels in the brain and that high-AS 126-expression genotypes accumulate with aging in normal controls [138]. Additionally, AS 126 mRNA levels are drastically decreased in LBD frontal cortices in comparison with controls [135]. Both findings suggest that AS126 plays a protective role in the aging brain.

#### 6.1.2. AS112

On the contrary, AS 112, that shows the shortening of the unorganized AS C-terminal, could be characterized by increased aggregation properties. Accordingly, isoform expression profiles obtained in frontal cortices of LB diseases revealed AS 112 overexpression, specifically in DLB [139].

Interestingly, initial perikaryal AS accumulation seems to be specifically constituted by AS112 [39,139]. AS112 lacks part of the unfolded protein C-terminal but conserves intact membrane-protein and protein-protein binding domains. Therefore, AS112 appears to be an aggregation-prone isoform with enhanced membrane-binding properties [39,139]. Additionally, the fact that aggregation of AS is preceded by its interaction with membranes [137] and that the small fraction of membrane-bound AS seeds the accumulation of the far more abundant cytosolic form [140], point to the AS112 membrane-protein interaction domain as a crucial element in early aggregation steps [39].

#### 6.1.3. AS98

Finally, since AS 98 preserves mainly an unchanged central region that contains aggregation-prone (amino acids 71–82) and toxic (amino acids 67–82, 72–82) sequences [48–50,141], it could be the most amyloidogenic and toxic AS isoform. Accordingly, AS 98 mRNA is overexpressed in LBD as well as Alzheimer disease [132].

### 6.2. Synphilin-1 alternative splicing

In addition to the differential expression of AS isoforms, alternative splicing of other genes with an important involvement in LBD pathogenesis seems also to be affected. Of these, synphilin-1 bears 8 isoforms as result of alternative splicing.

As a major AS interaction protein on one hand [116] and as a parkin substrate on the other [117,119,142], the main functional synphilin-1 domains are responsible for the interaction with them. They are variably shortened in the transcript variants synphilin-1A, 1B and 1C. The four shortest isoforms correspond to truncated, probably unfuntional proteins.

#### 6.2.1. Synphilin-1A

Synphilin-1A is an unusual splice variant of synphilin-1 [58]. It is characterized by a different start codon leading at the same time to a different initial reading frame. Moreover, synphilin-1A lacks the first 394 amino acids and contains additional, very protein specific, amino acid streches, one at the N-and the other on the C-terminus [58].

Synphilin-1A is an aggregation-prone and neurotoxic protein but forms nevertheless neuroprotective inclusions. Moreover, that synphilin-1A interacts with synphilin-1 as well as AS and localizes in LBs, suggesting that synphilin-1A performs its own, isoform-specific function [117,143]. In this context, synphilin-1A’s early involvement during LBD pathogenesis could be confirmed by our finding of synphilin-1A overexpression in PD together with its normal levels in pDLB [136].

Due to its intrinsic neurotoxicity, interaction changes between synphilin-1A and AS could be involved not only in in LB formation but also in dopaminergic neuronal death [66].

### 6.3. Parkin alternative splicing

So far, 7 different transcript variants (TV) of parkin have been identified. Parkin as one of E3-ubiquitin ligases [124], contains an ubiquitin-like as well as a ring-finger domain, both involved in ubiquitylation of a specific substrate. Parkin isoforms TV2 and TV3 show a shortened sequence between functional domains [144], possibly leading to changes in the efficiency of substrate recognition or ubiquitylation.

As a possible result, the shortened isoforms of synphilin-1 and parkin could present stronger interactions than normal-length proteins. The non-dissociation of synphilin-1 – parkin or synphilin-1 – AS complexes could play an important role during the protein aggregation that precedes LB formation. SNCA and synphilin-1 overexpression in PD frontal cortex, a region not yet affected by LB pathology, underlines this idea, and is further supported by the finding that synphilin-1 and parkin are early components of LBs [119,143].

### 6.4. Isoform expression profiles

Recently, we have obtaind isoform exression profiles composed by the 4 AS, 4 synphilin-1 and 7 parkin isoforms [136]. We have shown that the different LB diseases present their own isoform expression profiles in frontal cortex. Changes in protein-protein interactions resulting from the lack or overrepresentation of some of the isoforms could be responsible for diminution or gain of function. Since both parkin and synphilin-1 are involved in the proteasome mediated protein degradation, and an important proteasome dysfunction can be observed in synucleinopathies, one of the mechanisms leading to such a dysfunction could be the alteration of alternative splicing.

Moreover, the results strongly support the idea that the different LBD are characterized by their own molecular mechanisms of disease development. Furthermore, our data strengthen the hypothesis that different molecular mechanisms lead to the development of similar neuropathological changes. Elucidating these different mechanisms, as well as ascertaining the clinical diagnosis of LB diseases, will help in the development of appropriate and efficient treatments for each of them.

## Conclusions

A main characteristic of LBD, similar to other neurodegenerative conditions, is their genetic heterogeneity. Whereas multiple forms of familial PD are caused by mutations in at least seven different genes, sporadic cases that represent the majority of all LBD do not show a defined genetic profile. This observation, in addition to the alteration of multiple pathways during disease development, suggests the existence of more than one subtype of each disease where similar neuropathological changes are the result of different primary molecular mechanisms.

All patients with clinical diagnosis of PD or DLB, present LBs when analyzed in a post-mortem, neuropathologic study. The only exception are juvenile PD cases caused by mutations in the parkin gene. In contrast, LBs are also found in a relative high percentage of asymptomatic individuals [145,146]. These observations strongly indicate that some individuals are provided by protective mechanisms against LBD, but that LBs and their formation process are closely related to disease development. Although the sequence of brain area affectation is now well-known, neither the earliest mechanisms responsible for AS aggregation nor their chronological order have been determined. Indeed, the exact sequence of pathological alterations that finally lead to disease development remains unknown. Taken together these observations, it would seem that efficient treatments will differ in all PD and DLB groups, or even in patients.
